# Lysosomal Interaction of Akt with Phafin2: A Critical Step in the Induction of Autophagy

**DOI:** 10.1371/journal.pone.0079795

**Published:** 2014-01-08

**Authors:** Mami Matsuda-Lennikov, Futoshi Suizu, Noriyuki Hirata, Manabu Hashimoto, Kohki Kimura, Tadashi Nagamine, Yoichiro Fujioka, Yusuke Ohba, Toshihiko Iwanaga, Masayuki Noguchi

**Affiliations:** 1 Division of Cancer Biology, Institute for Genetic Medicine, Hokkaido University, Kita-ku, Sapporo, Japan; 2 Laboratory of Pathophysiology and Signal Transduction, Hokkaido University Graduate School of Medicine, Kita-ku, Sapporo, Japan; 3 Laboratory of Histology and Cytology, Hokkaido University Graduate School of Medicine, Kita-ku, Sapporo, Japan; National Institute of Dental and Craniofacial Research, United States of America

## Abstract

Autophagy is an evolutionarily conserved mechanism for the gross disposal of intracellular proteins in mammalian cells and dysfunction in this pathway has been associated with human disease. Although the serine threonine kinase Akt is suggested to play a role in this process, little is known about the molecular mechanisms by which Akt induces autophagy. Using a yeast two-hybrid screen, Phafin2 (EAPF or PLEKHF2), a lysosomal protein with a unique structure of N-terminal PH (pleckstrin homology) domain and C-terminal FYVE (Fab 1, YOTB, Vac 1, and EEA1) domain was found to interact with Akt. A sucrose gradient fractionation experiment revealed that both Akt and Phafin2 co-existed in the same lysosome enriched fraction after autophagy induction. Confocal microscopic analysis and BiFC analysis demonstrated that both Akt and Phafin2 accumulate in the lysosome after induction of autophagy. BiFC analysis using PtdIns (3)P interaction defective mutant of Phafin2 demonstrated that lysosomal accumulation of the Akt-Phafin2 complex and subsequent induction of autophagy were lysosomal PtdIns (3)P dependent events. Furthermore, in murine macrophages, both Akt and Phafin2 were required for digestion of fluorescent bacteria and/or LPS-induced autophagy. Taken together, these findings establish that lysosomal accumulation of Akt and Phafin2 is a critical step in the induction of autophagy via an interaction with PtdIns (3)P.

## Introduction

Intracellular degradation and recycling of proteins is carried out by an evolutionarily conserved process called autophagy [1]–[3]. The process of autophagy involves the sequestering of cytosolic proteins or organelles within double–membrane vesicles derived from the lysosome which is then followed by degradation and/or recycling of the protein molecules.

Recently attention has turned to cross-talk regulation between anti-apoptosis and induction of autophagy [4], [5]. Serine threonine kinase Akt, also known as Protein Kinase B, regulates numerous cellular processes, including anti-apoptosis, proliferation, cell cycle, cytoskeletal organization, vesicle trafficking, and glucose transport [6], [7].

The PI3K-Akt-mTOR pathway, which mediates anti-apoptotic signaling, is suggested to have an important role in the regulation of autophagy in mammalian cells [4], [8], [9], [10]. Nevertheless, the precise molecular mechanism by which Akt signal integrates into the regulation of autophagy is unknown.

In this study we demonstrate that lysosomal accumulation of an Akt-Phafin2 complex is critical in the induction of autophagy and is mediated by an interaction with lysosomal PtdIns(3)P. An Akt-Phafin2 functional interaction not only demonstrates a molecular role for the PI3K-Akt signaling pathway in the regulation of autophagy, but also explains how 3-MA (3-methyladenine), a widely used autophagy inhibitor, suppresses autophagy in mammalian cells.

## Results

### Phafin2 interacts with Akt in mammalian cells

**Figure 1:**
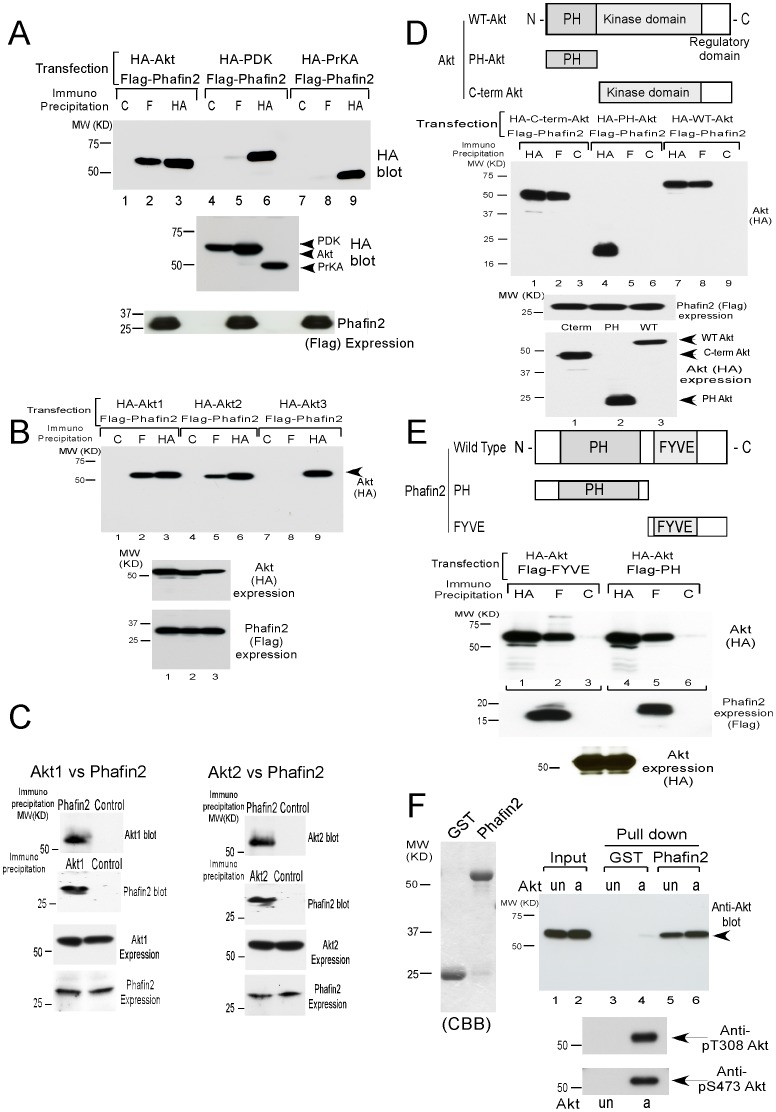
Phafin2 associates with Akt in mammalian cells. **A**. Flag-Phafin2 interacted only with HA-Akt, but not with HA-PDK1 or HA-PrKA. Similar levels of expression of HA-Akt, PDK1, PrKA, and Flag-Phafin2 were shown by immunoblot (HA = anti-HA antibody; F = anti-Flag antibody; C = Control antibody). **B**. Flag-tagged Phafin2 interacted with HA-tagged Akt1 (lane 1–3) and Akt2 (lane 4–6), but not with Akt3 (lane 7–9). Similar levels of three Akt isoforms and Phafin2 were expressed (lower panels). **C**. Endogenous Akt1 and Akt2 interacted with Phafin2 in HT1080 cells by co-immunoprecipitation assays. Expression of Akt isoforms and Phafin2 were shown (lower panels). **D**. The C-terminal Akt kinase domain is the binding domain for Phafin2 interaction in co-immunoprecipitation assays. Expression of the Flag-Phafin2 and HA-Akt subfragments were shown (lower panels). **E**. Structural features of Phafin2 used in this study are shown. Phafin2 consisted of N-terminal PH domain and C-terminal FYVE domain. Both PH domain and FYVE domain interacted with Akt in co-immunoprecipitation assays. Expression of Akt and Phafin2 subfragments were shown (lower panels). **F**. Interaction between recombinant Phafin2 and Akt was verified in GST pull-down assays. Recombinant active (a) or unactive (un) Akt was incubated with GST-Phafin2 beads and subsequently resolved onto SDS-PAGE and detected by immunoblotting using anti-Akt antibody (right panel). Levels of phosphorylation of active Akt (a) and unactive Akt (un) were shown (lower panels).

Utilizing a yeast two-hybrid screening approach with Akt2 as bait, a stable interaction with Phafin2 (also known as EAPF or PLEKHF2) [11] was identified. Phafin2 is a lysosomal protein consisting of 249 amino acids with a unique structure containing both an N-terminal PH (pleckstrin homology) domain and C-terminal FYVE (Fab 1, YOTB, Vac 1, and EEA1) domain (see Fig. 1E) [12]–[15].

Akt, but not PDK1 or PrKA, interacted with Phafin2 in co-immunoprecipitation assays in 293T cells (**Fig. 1A**). Three Akt isoforms (Akt1, Akt2, and Akt3) are present in the human genome, with more than 85% homologies at the amino-acid level [7]. In co-immunoprecipitation assays using 293T cells, Akt1 and Akt2, but not Akt3 interacted with Phafin2 (**Fig. 1B**). In HT1080 cells, an endogenous interaction of Akt1 and Akt2 with Phafin2 was confirmed via co-immunoprecipitation assays (**Fig. 1C**). Two isoforms of Phafin are reported in the human and mouse genome, containing similar structures with PH and FYVE domains. Because Phafin2 was identified as a binding partner for Akt2 in yeast two-hybrid screening, we focused our investigation on Phafin2.

Akt contains an N-terminal PH (Pleckstrin Homology) and a C-terminal catalytic kinase domain. Immunoprecipitation experiments with the different Akt domains identified a stable interaction between Phafin2 and the C-terminal domain of Akt (**Fig. 1D**). To define the domains within Phafin2 that interact with Akt, Phafin2 subfragments were generated in mammalian expression vectors. The interaction between Phafin2 and Akt was mediated through both the FYVE domain and the PH domain of Phafin2 (**Fig. 1E**).

Because phosphorylation plays a key role in protein binding of intracellular molecules, we verified the phosphorylation-dependent interactions of Phafin2 with Akt. In a GST pull-down assays, both the active and unactive Akt (Upstate biotechnology) interacted equally with Phafin2 (**Fig. 1F**). These results demonstrate the Phafin2-Akt interaction is not a phosphorylation dependent event.

### Phafin2 displays lysosomal accumulation with Akt after induction of autophagy

As Phafin2 is a lysosomal protein [11], we examined whether and how Phafin2 co-localized with Akt following autophagy induction by rapamycin or HBSS treatment.

**Figure 2:**
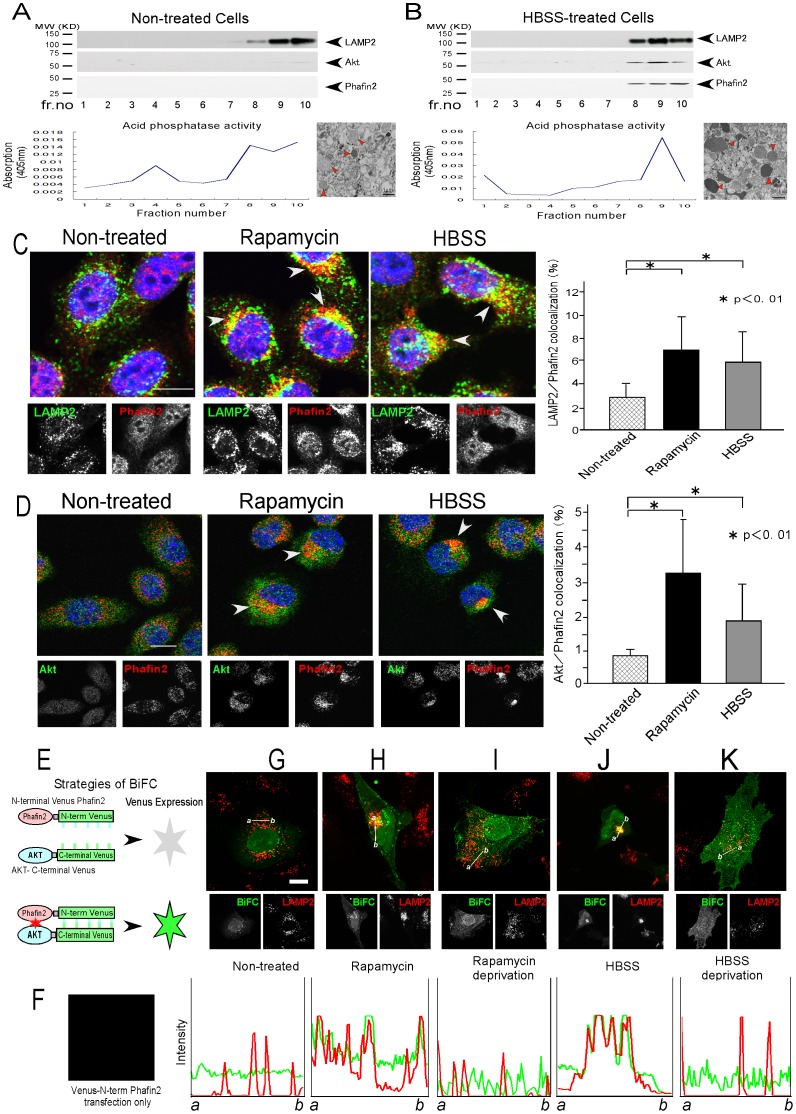
Phafin2 displays accumulation at the lysosome with Akt after induction of autophagy. **A–B**. HT1080 cells were cultured in DMEM supplemented with 10% FBS (**A**, non-treated) or HBSS (**B**), and sucrose gradient subcellular fractionation was conducted and resolved onto SDS-PAGE and immunoblotted by indicated antibodies. Acid phosphatase activity of each fraction was measured by optical absorption using Acid Phosphatase Assay kit (Sigma) and TEM examination of the crude fraction of lysosome before and after HBSS treatment to induce autophagy was also shown underneath. **C–D**. Cells were cultured in complete media (non-treated, left panels), treated with rapamycin (middle panels) or HBSS (right panels) to induce autophagy. HeLa cells were fixed and immunostained by anti-LAMP2 (green) along with anti-Phafin2 (red) antibodies (C). J774.1 cells were immunostained by anti-Akt (green) along with anti-Phafin2 (red) antibodies (D). Colocalized areas in the merged view were indicated by white arrow heads. The quantification of the colocalized area of Phafin2 with lysosome (C) or Akt with Phafin2 (D) per cell (%) was shown as a bar graph with statistical analysis by Mann Whitney U-test (right side). White scale bar represents 10 µm. The results were consistent between three independent experiments. **E–K**. Using BiFC, localization of Akt and Phafin2 with lysosome was examined (**E**). pCAGGS-VN-Phafin2 alone (F) or both pCAGGS-VN-Phafin2 and pCAGGS-VC-Akt2 (G–K) were transfected into HeLa cells and cultured in normal condition (**G**) or treated with rapamycin (**H**) and subsequent removal of rapamycin (**I**). For HBSS treated cells, the culture media was replaced with HBSS for 4 hours (**J**). HBSS were subsequently replaced with complete media (DMEM) with 10% FBS (**K**). The cells were fixed and immunostained with anti-LAMP2 antibody and visualized by confocal microscopy. Fluorescence intensities of BiFC (green) and AlexaFluor594 (red) along the line (*a–b*) were plotted underneath. White scale bar represents 10 µm.

Sucrose fractionation experiments demonstrated that Phafin2 co-existed with Akt in the same lysosomal fraction only after induction of autophagy (**Fig. 2A and B**, upper panels). Transmission Electron Microscope (TEM) further confirmed the fractions purified by sucrose gradient contained early and late lysosomes (**Fig. 2A and B**, lower panels).

Confocal microscopy studies suggested that Phafin2, which is normally diffusely spread in the cytosol, became localized in the lysosomal fractions (as detected by LAMP2, **Fig. 2C**) after the induction of autophagy. Lysosomal accumulation/co-localization of Akt with Phafin2 (**Fig. 2D**) or Akt with lysosome (LAMP2) was augmented after the induction of autophagy. Finally, FRET assays demonstrated the interaction of Akt with Phafin2 was enhanced after induction of autophagy (**fig. S1**).

Bimolecular fluorescence complementation (BiFC) analysis produces a specific fluorescence upon the interaction between two protein components, making it amenable to intracellular imaging techniques [16] (Fig. 2E). Individual expression of Akt or Phafin2 tagged with either the N- or C-terminal fragment of Venus yielded no background signal (**Fig. 2F**). Under normal conditions, Akt interacts with Phafin2, and localized in the nucleus and cytoplasm (**Fig. 2G**). However, following the induction of autophagy, it relocated to associate with the perinuclear lysosome as determined by co-localization with anti-LAMP2 staining (**Fig. 2H and J**). Moreover, this accumulation of the Akt-Phafin2 complex following autophagy induction was reversible and the aggregates were dispersed following the removal of either rapamycin or HBSS treatment (**Fig. 2I and K**). Collectively, these observations provide evidence that formation of the Akt-Phafin2 complex on the lysosome is augmented after the induction of autophagy.

### Interaction of PtdIns(3)P with Phafin2 determines lysosomal localization of the Akt-Phafin2 complex

Phafin2 contains a unique structure with an N-terminal pleckstrin homology (PH) domain and a C-terminal FYVE domain, both of which are known binding motifs for PtdIns(3)P [12], [14], [15] (see Fig. 1E). PtdIns(3)P, which is abundantly present on the lysosome, plays a critical role in autophagy [17]. Because of these observation we next investigated the role of PtdIns(3)P in the lysosomal accumulation of Akt-Phafin2 protein complex during autophagy.

**Figure 3:**
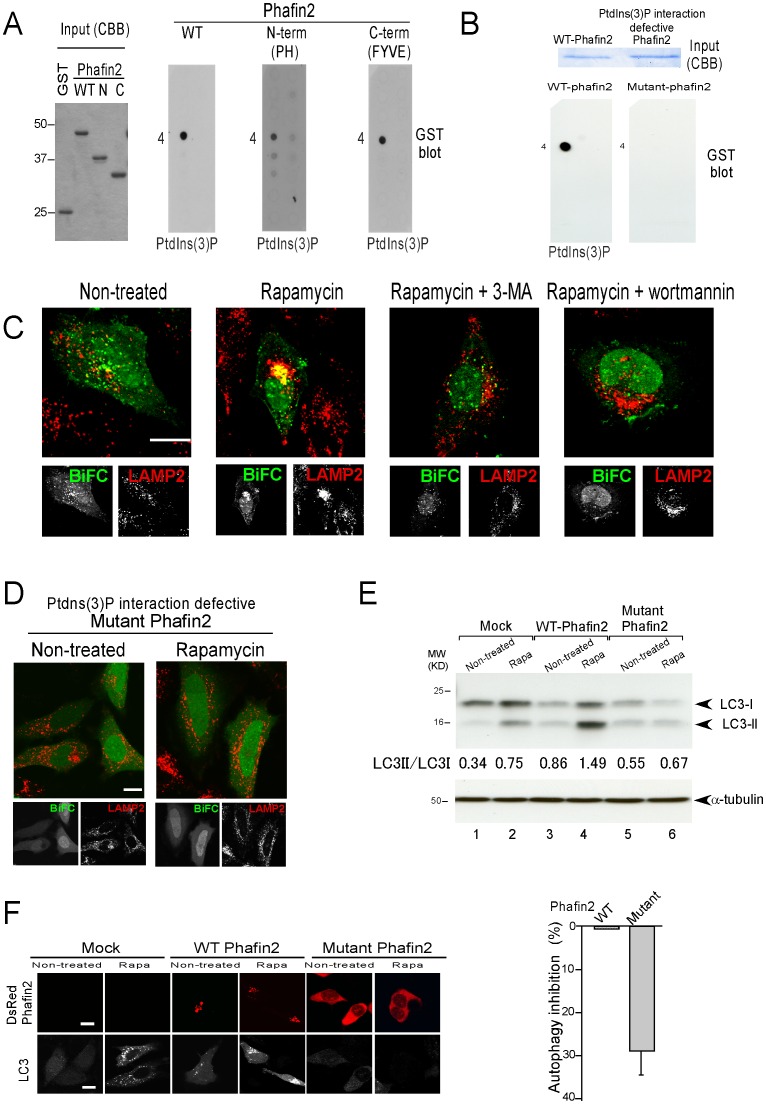
The interaction of the PtdIns(3)P with Phafin2 determines the lysosomal localization. **A**. PIP strips were incubated with GST-Phafin2 WT, PH, or FYVE and the binding ability was examined by GST immunoblot. A comparable amount of recombinant wild type, N-term (PH domain), or C-term (FYVE domain) of Phafin2 was used (left panel). **B**. PtdIns(3)P interacting defective-Phafin2 (R53C, R171A, and R172A) failed to bind to PtdIns(3)P on PIP strip (right panel). **C**. BiFC analysis, 3-MA or wortmannin treatment abrogated the accumulation of the Akt-Phafin2 protein complexes at perinuclear lysosome after rapamycin treatment. **D**. Mutant Phafin2 displayed no accumulation of perinuclear lysosome after rapamycin treatment (right panels). **E**–**F**. Mutant Phafin2 failed to induce autophagy determined by decreased intensity of LC3-II band (**E**, lane 6). The percentage of autophagy inhibition was 29.3±7.50% out of three independent experiments. The observation was consistent, as determined by the absence of GFP-LC3 puncta using confocal microscopy (**F**, lower right side panels). Note that ectopic expression of wild type Phafin2 modestly enhanced autophagy induction determined by LC3-II blot (E, lane4 upper panel) and LC3 puncta (F. lower panels). White scale bar represents 10 µm.

Using PIP strips, full-length Phafin2, Phafin2-PH domain, and Phafin2-FYVE domains interacted preferentially with PtdIns(3)P (**Fig. 3A** and **fig. S2**), consistent with previous research [18]. Phafin2 contains an RRHHCR motif within the FYVE-domain and KPKARQF motif within the PH-domain, which are putative interaction motifs for PtdIns(3)P [12]–[14]. We created a PtdIns(3)P interaction-defective mutant Phafin2 (R53C, R171A, and R172A, hereafter referred to as mutant Phafin2) for BiFC [19]. Mutant Phafin2 cannot interact with PtdIns(3)P (**Fig. 3B**) and displayed no lysosomal accumulation of the Akt-Phafin2 complex after induction of autophagy (**Fig. 3D**). Although the mutant Phafin2 did not bind to PtdIns(3)P it retained the ability to bind with Akt, demonstrated by co-immunoprecipitation (**Fig. S3**). The PI3K-Akt-mTOR pathway mediates anti-apoptotic signaling and is demonstrated to play an important role in the regulation of autophagy in mammalian cells [2], [4], [8]–[10]. To better define the role of PtdIns(3)P in autophagy and the interaction with Akt-Phafin2, complex formation was followed by BiFC after treatment with either 3-MA (class III PI3K inhibitor and inhibitor of autophagy [21]) or wortmannin [class I PI3K inhibitor [9]]. Both treatments blocked the lysosomal accumulation of the Akt-Phafin2 complex after the induction of autophagy (**Fig. 3C**). The results suggest the interaction of PtdIns(3)P with Phafin2 is necessary for the induction of autophagy.

After rapamycin treatment to induce autophagy, wild type Phafin2 displayed a granular pattern in the cytosol during BiFC analysis (**Fig. 2E–K** and **Movie S1**). In contrast, this was not observed with the mutant Phafin2 (R53C, R171A, and R172A), which did not bind to PtdIns(3)P (Fig. 3B), (**Fig. 3D** and **Movie S2**). More importantly, mutant Phafin2 failed to induce autophagy in LC3 immunoblot and GFP LC3 puncta (**Fig. 3E and 3F**). These results suggest the interaction of PtdIns(3)P with Phafin2 is necessary not only for the lysosomal localization of the Akt-Phafin2 complex, but also for the induction of autophagy.

### Both Phafin2 and Akt are required for the induction of autophagy and the elimination of fluorescent bacteria

**Figure 4:**
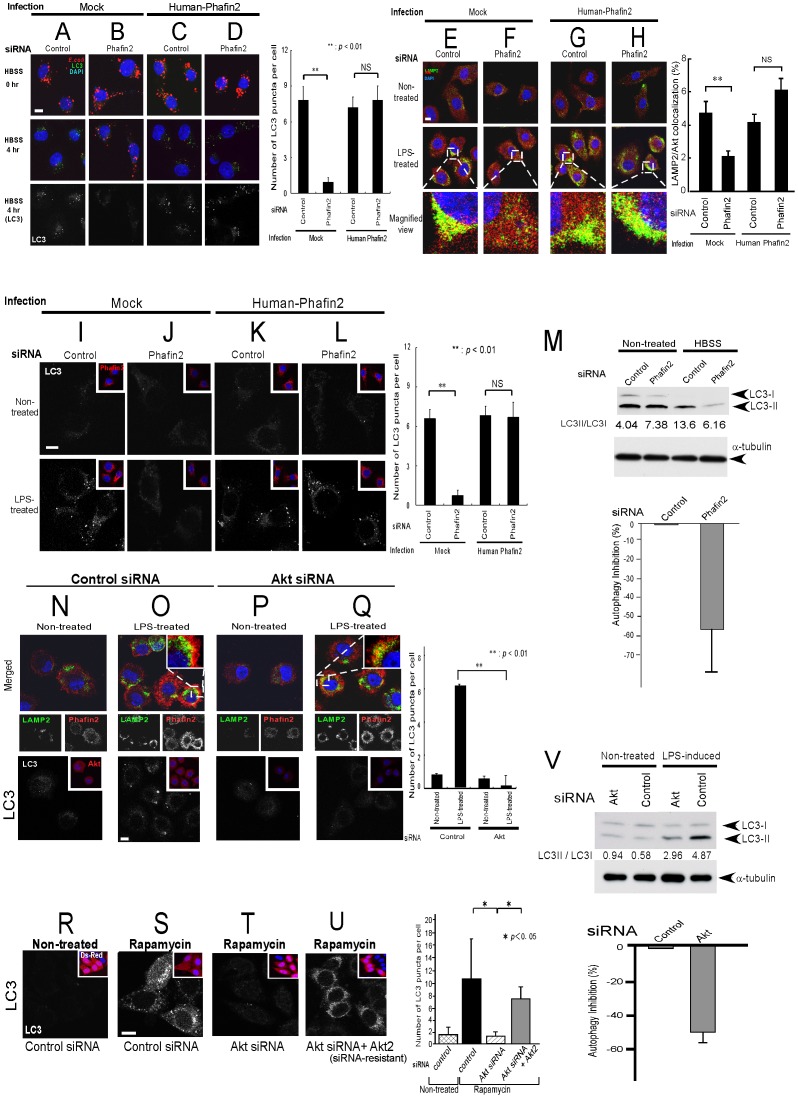
Presence of both Akt and Phafin2 are required for induction of autophagy. **A–D**. Phafin2-siRNA transfected macrophages showed no inhibition on initial uptake of fluorescent bacteria (**A–B**, top panels). However, after HBSS treatment to induce autophagy, Phafin2-siRNAs transfected cells inhibited not only elimination of fluorescent bacteria (**A–B**, middle panels), but also induction of autophagy (**A–B**, bottom panels), which is reversible by re-introduction of human Phafin2 (**C–D**, bottom panels). Note that human Phafin2 is resistant for mouse Phafin2-siRNA. Quantification of LC3 puncta per cell with statistical analysis by Student's *t*-test is shown on the right side. White scale bar represents 10 µm. **E–H**. Using J774.1 murine macrophages, LPS-induced lysosomal accumulation of Akt was eliminated by Phafin2-siRNAs (**E–F**), which is associated with inhibition of autophagy (**I–J**, lower panels). The observations are reversible by re-introduction of human Phafin2 (**G–H** and **K–L**, lower panels). Quantification of the percent of colocalization area of Akt with LAMP2 with statistical analysis by Student's *t*-test is shown on the right side. **I–L**. Phafin2-siRNA transfected macrophages inhibited LPS-induced autophagy determined by LC3 pancta with Phafin2 expression shown in inset (bottom panels, compare **I** and **J**). Inhibition of autophagy by Phafin2-siRNAs can be reverted by re-introduction of human Phafin2, which is resistant for mouse Phafin2-siRNA (bottom panels, compare **K** and **L**). Quantification of LC3 puncta per cell with statistical analysis by Student's *t*-test is shown on the right side. **M**. LC3 immunoblot by the introduction of Phafin2-siRNA were shown. The percentage of autophagy inhibition out of three independent experiments was 53.3±21.4%. **N–Q**. Akt-siRNA (fig. S5) transfected macrophages retained LPS-induced lysosomal translocation/accumulation of Phafin2 (upper panels). Akt-siRNA, however, inhibited LPS-induced autophagy determined by LC3 pancta with Akt expression shown in inset (bottom panels, compare **O** and **Q**). Number of GFP-LC3 puncta per cells with statistical analysis by Student's *t*-test were shown as a bar graph. **R–U**. Akt-siRNA (fig. S5) transfected HeLa cells failed to induce autophagy determined by LC3 puncta on Ds-Red positive cells (inset). Further, inhibition of autophagy by Akt-siRNAs can be reversed by re-introduction of siRNA-resistant human Akt2 in HeLa cells (**U**). Number of GFP-LC3 puncta per cells with statistical analysis by Student's *t*-test were shown as a bar graph. **V**. LC3 immunoblot by the introduction of Akt siRNA were shown. The percentage of autophagy inhibition (shown in the bar graph) was 45.4±11.2% out of three independent experiments.

In addition to its role as a disposal system of intracellular protein, autophagy is thought to play a role as a defensive mechanism against intracellular pathogens [3], [20]. Phafin2-siRNA transfected J774.1 mouse macrophages (**fig. S4**) show normal uptake and elimination of fluorescent bacteria (**Fig. 4A–B**, top panels). However, following HBSS treatment to induce autophagy, Phafin2-siRNAs transfected cells failed to eliminate fluorescent bacteria (**Fig. 4A–B**, middle panels). This is associated with the inhibition of induction of autophagy determined by the absence of LC3 puncta (**Fig. 4A–B**, bottom panels) and is reversible by the re-introduction of human Phafin2, which is resistant to inhibition by the mouse specific Phafin2-siRNA (**Fig. 4C–D**).

Lipopolysaccharide (LPS) is known to induce autophagy in macrophages as part of their role in mediate innate immunity [20]. Using J774.1 murine macrophages, LPS-induced lysosomal translocation/accumulation of Akt was eliminated by Phafin2-siRNAs (**Fig. 4E–F**, lower panels), and the induction of autophagy (**Fig. 4I–J**, lower panels). This could again be reversed via the re-introduction of the human Phafin2 (**Fig. 4K–L**, lower panels), confirming that Phafin2 is required for lysosomal translocation of Akt (**Fig. 4G–H**). Furthermore, LC3 immunoblot was used to confirm that Phafin2 is required for the induction of autophagy (**Fig. 4M**).

In contrast, Akt-siRNA (**fig. S5**) transfected macrophages retained the LPS-induced translocation/accumulation of Phafin2 on the lysosome (**Fig. 4Q**, upper panels, compare with Fig. 4F), but failed to induce LPS-initiated autophagy (**Fig. 4N–Q**, bottom panels). Moreover, Akt-siRNA inhibited the induction of autophagy in HeLa, which could be reversed by the re-introduction of siRNA-resistant human Akt2 (**Fig. 4R–U**). LC3 immunoblot confirmed Akt-siRNA inhibition of LPS-initiated autophagy (**Fig. 4V**).

## Discussion

**Figure 5:**
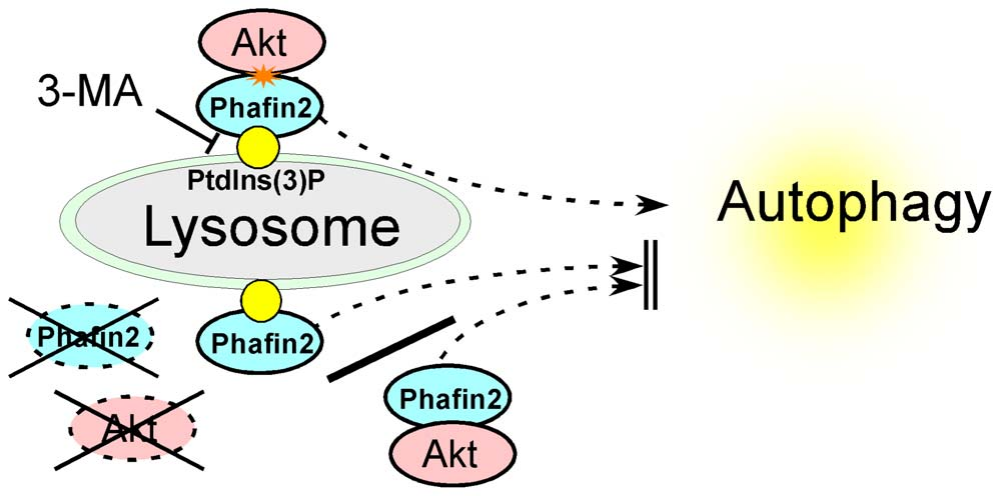
Lysosomal interaction of Akt with Phafin2 is a critical step to induce autophagy. PI3K-Akt pathway that mediates anti-apoptotic signal is suggested to play an important role in the regulation of autophagy in mammalian cells. However, molecular mechanisms by which Akt signal regulates autophagy are largely unknown. In this study, we demonstrated that the presence of both Akt and Phafin2 on the lysosome is critical in the induction of autophagy via interaction of lysosomal PtdIns(3)P in mammalian cells. Akt-Phafin2 functional interaction not only clarifies the molecular basis of the PI3K-Akt signaling pathway in the regulation of autophagy, but also shows how 3-MA (3-methyladenine), a widely used autophagy inhibitor, inhibits autophagy in mammalian cells at molecular levels. It has been suggested that Akt activation prevents induction of autophagy, however, the roles of Akt in the regulation of autophagy induction is not clear. Hence, the current studies add a new twist to the molecular regulation of autophagy via PI3K-Akt signaling pathways in mammalian cells.

Although PI3K-Akt-mTOR pathway is reported to play an important role in the regulation of autophagy [4], [8], [9], [10], [21], [22], the molecular basis by which Akt controls the induction of autophagy remains unknown. In this study, we demonstrate that Phafin2 physically interacts with Akt and translocates to the lysosome upon induction of autophagy (**Fig. 5**). This study demonstrates the presence of Akt in the lysosome is required for the induction of autophagy. In co-immunoprecipitation assays, both Akt1 and Akt2, but not Akt3 interacted with Phafin2. In contrast to wild type MEF cells, Akt1-/-, Akt2-/- (DKO) MEF cells failed to induce autophagy after rapamycin treatment [24]. However, the re-introduction of HA-Akt2 but not Akt1 restores the ability to induce autophagy as determined by the presence of LC3 puncta. These results show that the lysosomal localization of the Akt2-Phafin2 complex is likely to play an important role in the induction of autophagy. This was supported by our initial observation that the C-terminal Akt truncation mutant, which lacks the Akt Ser473 phosphorylation site but retains the ability to interact with Phafin2, failed to induce autophagy. Moreover, the levels of Akt phosphorylation and phosphorylated Akt substrate(s) appear to be increased in the lysosome after induction of autophagy by both rapamycin and HBSS treatment. These observations suggest that Phafin2 relocates cytosolic Akt to the autophagosome/autolysosome to regulate autophagy. Further studies will clarify whether previously undetermined Akt substrate(s) may regulate autophagy at autophagosome/autolysosome.

PI3K (Phosphoinositide 3 Kinase) is a family of lipid kinases which phosphorylate the 3′OH group of the inositol rings of inositol phospholipids [23]. Three classes of PI3K (class IA, IB, II, and III PI3Ks) are defined by their distinct substrate preferences. Class I PI3Ks are responsible for the production of Phosphatidylinositol 3-phosphate [PtdIns(3)P], Phosphatidylinositol (3,4)-bisphosphate (PI(3,4)P_2_), and Phosphatidylinositol (3,4,5)-trisphosphate [PI(3,4,5)P_3_] [23]. Class III PI3Ks produces only PtdIns(3)P from PI by forming a heterodimer with the catalytic (Vps34) and regulatory (Vps15/p150) subunits.

In this study, we demonstrate that 3-MA (3-methyladenine, class III PI3K inhibitor) treatment, which inhibits the generation of PtdIns(3)P, inhibited the lysosomal accumulation of the Akt-Phafin2 complex (Fig. 3C). Moreover, PtdIns(3)P interaction defective mutant Phafin2 showed no lysosomal accumulation of Akt-Phafin2 complex (Fig. 3D). Upon Akt activation, PtdIns(3,4,5)P_3_ is increased at the plasma membrane by primarily class I PI3K activity and inhibits autophagy. However, upon autophagy induction, PtdIns(3)P, which recruits Phafin2 bound Akt to the lysosome, is increased by class III PI3K activity, facilitating the induction of autophagy. Unlike Phafin2-PH and FYVE domain, both of which recognize PtdIns(3)P (see Fig. 3A), Akt-PH domain preferentially binds to PtdIns(3,4)P_2_ or PtdIns(3,4,5)P_3_ [12]. This unique structural feature of the Akt-Phafin2 complex may contribute to the stoichiometry of the lysosomal localization of the Akt-Phafin2 complex upon autophagy induction [10].

Identification of an Akt-Phafin2 interaction not only clarifies the previously unknown molecular role of the PI3K-Akt signaling pathway in the regulation of autophagy, but also suggests that Akt-Phafin2 interaction is likely to be the molecular target for inhibition of autophagy by 3-MA (3-methyladenine, class III PI3K inhibitor), a widely used pharmacological inhibitor of autophagy [22], [24], [25].

## Materials and Methods

### Ethics statement

Cell lines (HT1080, 293T, HeLa, J774.1) used in this study were purchased from ATCC.

No animals were used in conduction with this study.

This work does not involve any human participants.

### Yeast Two-Hybrid screening

Yeast two hybrid assays were performed as previously described [26], [27]. Y190 cells (Clontech) were transformed by the lithium acetate method with the “bait plasmid” (human Akt2/pAS2-1) according to the manufacturer's protocol. β-gal positive clones were mated with Y187 yeast (MATα gal4 gal80 his3 trp1–901 ade2–101 ura3–52 leu2–3,-112 met2URA3::GAL–.lacZ) carrying pAS1-CYH2 with or without an insert or with SNF-1 or p53 to determine the specificity for the interaction. Two independent clones were identified that encode full length cDNA of human Phafin2.

### Co-immunoprecipitation experiments

Co-immunoprecipitation experiments were performed as previously described [26], [27]. In brief, 293T cells (ATCC) were co-transfected with a total of 7.5 µg of indicated plasmids per 10cm dish. Seventy two hours after transfection, cells were washed twice with ice-cold PBS and lysed with ice-cold Brij97 lysis buffer with proteinase inhibitors (Leupeptin, and AEBSF). Lysates were precleared with protein G/protein A sepharose beads mixture (50% v/v, GE healthcare) for 1 hr., immunoprecipitated with anti-HA or anti-Flag antibody (or other indicated antibodies) with mouse IgG as a control. The samples were run on SDS-PAGE, immunoblotted with indicated antibodies and detected using ECL.

### Interaction of recombinant Phafin2 with active and unactive Akt

A comparable amount of either GST control or GST-wild type Phafin2 recombinant protein were generated according to the manufacturer's protocol (GE healthcare) and shown by CBB-stained gel. Recombinant active (a) or unactive Akt (un) (#14-276 active Akt1/PKBα, #14-279 “unactive” Akt1/PKBα, Upstate Biotech, USA) were incubated with either GST-Phafin2 or GST protein as a control in [1% Triton buffer, 50 mM Tris HCl (pH 7.5), 150 mM NaCl] for 5 min. at 4°C. The resulting samples were washed by three times,resolved onto SDS-PAGE and immunoblotted using anti-Akt antibody (Cell Signaling). The levels of phosphorylation of the recombinant active (a) or unactive Akt (un) were verified by immunoblotting using anti-phospho Ser473 Akt (Cell Signaling, #4051S) or anti-phospho Thr308 Akt (Cell Signaling, #9275S) antibodies as indicated and detected using ECL.

### Lysosomal fractionation of HT1080 cells

HT1080 cells (2.5×10^8^ cells, ATCC) were cultured in DMEM (Sigma, #D5796) supplemented with 10% FBS at 37°C (control, left panels). The cells were washed with PBS three times and subsequently treated with HBSS (Hank's Balanced Salt Solution, Gibco, 14025-092) for 4 hours (HBSS-treated cells, right side panels), harvested, then rinsed twice with ice-cold PBS. The lysosomal enriched fraction was purified using Lysosome Isolation Kit (LYSISO1, Sigma), resolved onto SDS-PAGE, and immunoblotted by indicated antibodies and detected using ECL. Acid phosphatase assay was conducted using Acid Phosphatase Assay kit according to the manufacturer's instruction (Sigma, CS0740-1KT). Briefly, after fractionation, each sample was transferred to new tube and substrate solution was added to the tube, vortexed, and incubated for 5 min at 37°C. Next stop solution (0.5N NaOH) was added and the absorption was measured at 405 nm using DU800 spectrophotometer (Beckman Coulter). For electron microscopic observation (TEM), the subcellular sucrose fractionations (non-treated or HBSS-treated samples) were centrifuged and fixed for 2 hr via addition of 2.5% glutaraldehyde in 0.1M phosphate buffer (pH 7.4). The fixed cell-pellets were detached from tubes, post-fixed in 1% osmium tetroxide, dehydrated, and embedded in Epon. Ultrathin sections were prepared and stained with both uranyl acetate and lead citrate for observation under an electron microscope (H-7100; Hitachi, Tokyo)

### Confocal microscopy

HeLa cells (ATCC) or J774.1 cells (ATCC) were cultured in DMEM supplemented with 10% FBS, then treated with 100 ng/ml of LPS (Invivogen) for 16 hours, 10 µM rapamycin (Sigma) for 4 hours, or washed with PBS three times and subsequently incubated in HBSS for 4 hours. The cells were fixed with 3.7% formaldehyde solution and immunostained with indicated combination of anti-Akt antibody (CST, #2966 or #9272), anti-Phafin2 antibody (Santa Cruz, sc-87358, abcam, ab56098, or home made), and anti-LAMP2 antibody (abcam, ab25631 or ab37024). The cells were stained with DAPI (4′,6-diamidino-2-phenylindole, blue, Sigma) and observed using confocal microscopy (FLUOVIEW FV1000-D, Olympus). Co-localized area per cell (%) was quantitated by NIH imageJ and shown as a bar graph with statistical analysis via Mann-Whitney U test. The white scale bar represents 10 µm. The results were consistent between three independent experiments.

### BiFC experiment

BiFC (bimolecular fluorescence complementation) analysis was performed as previously described [16], [28]. N-terminal Venus (cDNA for Venus, a variant of the yellow emitting mutant, YFP) fused Phafin2 in pCAGGS-VN (pCAGGS-VN-Phafin2) and C-terminal Venus fused to Akt2 (pCAGGS-VC-AKT2) were generated. To obtain human Akt2 in pCXN2-Venus CT vector, the internal NotI site of wild type human Akt2 cDNA was first mutated by Quik change (Agilent technologies) using the following primers: (HD513: GATGATGTGCGGACGCC TGCCCTTC and HD517: GAAGGGCAGGCGTCCGCACATCATC, substituted nucleotide within NotI site was underlined) and the resulting NotI mutated human Akt2 was subcloned into NotI and XhoI sites of the pCXN2-Venus CT vector (Akt C-term Venus). Human full-length (WT) Phafin2 in pCAGGS-Venus-NT vector was generated by PCR and subcloned into pCAGGS-Venus-NT vector (N-term Venus Phafin2) [19], [28]. Human Phafin2, in which three critical amino acids for mediating the interaction of PtdIns(3)P were altered was made using Quik Change (Agilent technologies) site directed mutagenesis at R53C (KPKARQF→KPKACQF), R171A, and R172A ( RRHHCQR→AAHHCQR). Wild type and the resulting mutant plasmids were transfected into HeLa cells by PEI [26]. The cells were plated on a 12-mm-diameter glass-base dish (Iwaki, 3911-035) in DMEM supplemented with 10% FBS. Twenty four hours after the transfection, cells were pre-treated for 1 hr with either 2 mM 3-MA (3-methyladenine, Sigma) [24]or 100 nM wortmannin (Sigma). The cells were subsequently treated with 10 µM rapamycin (Sigma) in DMEM supplemented with 10% FBS, or washed with PBS three times and subsequently incubated in HBSS (GIBCO) in the presence of 3-MA or wortmannin for 4 hours at 37°C. The cells were fixed with 3.7% formaldehyde solution and imaged as previously described [28]. Simultaneous visualization of colocalization between lysosome (LAMP2, red) and BiFC (Venus, green) by double color imaging was shown. Fluorescent intensities of BiFC (green) and LAMP2 (red) of the lines from *a* to *b* of each panel were shown underneath. The white scale bar represents 10 µm. The results were consistent between two independent experiments.

### Binding activity of Phafin2 to PIPs using PIP strips

Equal amount of either GST-control or GST-WT, N-term, or C-term Phafin2, PtdIns(3)P interaction defective-Phafin2 mutant (R53C, R171A, and R172A), and GST-WT, were first generated according to the manufacturer's protocol (GE healthcare). Twenty five nanograms of the indicated recombinant protein in 3% blocking buffer were incubated with PIP-STRIPS (Echelon Bioscience, P-6001) for 2 hr at room temperature and subsequently immunoblotted using anti-GST antibody (GE healthcare, #27-4577-01).

### Akt or Phafin2 siRNA experiments

J774.1 cells were transfected with 100 nM siRNA which is specific for firefly luciferase (control, Wako Nippon GENE), Phafin2 (Phafin2, MSS231003: Mouse Stealth Select RNAi: Plekhf2 Stealth Select RNAi™ 3 siRNA, Invitrogen) or Akt-siRNA (Cell Signaling, 6211, [29]) using CUY21 Pro-vitro (NEPAGENE Co. Ltd). Seventy two hours later, the siRNA-transfected cells were analyzed.

For re-introduction of Phafin2, retroviral vector pBabe-puro or the construct containing human Phafin2 was transfected into Phoenix-Eco cells. Infectious retroviruses in culture supernatants were harvested forty eight and seventy two hours after transfection. J774.1 cells were cultured with the viral supernatants for 24–72 hours after Phafin2 siRNA transfection.

For re-introduction of Akt2, pCMV6-Akt-siRNA resistant human Akt2 was generated by Quik change (Agilenttechnologies) using the following pair of primers: [HD 581: 5′-GATGTGCGGCCGCCTACCGTTT TACA ACCAGGACCACG-3′ and HD582: 5′-CGTGGTCCTGGTTGTAAAACG GTAGGC GGCCGCACATC-3′]. Mutated nucleotides were underlined [29]. HeLa cells (ATCC) were transfected with 100 nM Akt-siRNA (Cell Signaling, #6211) or firefly siRNA (Control, Wako Nippon GENE) plus 3 µg/well of pCMV6-Akt-siRNA-resistant human Akt2 or pBluescript (Agilent technologies). Seven micrograms/well of pDsRed-Express-N1 (Clontech) was simultaneously transfected as verification of siRNA delivery. Seventy two hours later, the cells were treated with Premo™ Autophagy Sensors (LC3B-GFP) for 16 hours [30] and analyzed for the presence of LC3 puncta using confocal microscopy.

### HBSS-induced *E.coli* elimination experiment

Phafin2 siRNA and/or retroviral human Phafin2-introduced J774.1 cells were treated with Premo™ Autophagy Sensors (LC3B-GFP) for 16 hours [30]. The cells were then incubated with Alexa Fluor 594-killed *E.coli* (E-23370, *Escherichia coli* Bioparticles®, Molecular Probes) at 10 µg/ml for 1 hour at 37°C, washed 3 times with pre-warmed PBS and incubated in HBSS for 4 hr to induce autophagy. Cells were fixed with 3.7% formaldehyde immediately after 4 hr incubation in HBSS. The cells were subsequently stained with DAPI (4′, 6-diamidino-2-phenylindole, blue, Sigma) and observed using confocal microscopy (FLUOVIEW FV1000-D, Olympus). The white scale bar represents 10 µm. LC3 puncta per cells were counted in 20 cells and shown as a bar graph. Statistical analysis was conducted via Student's *t*-test.

### LC3 immunoblot and GFP-LC3 puncta

The cells were harvested, washed 3 times with ice-cold PBS and lysed in Brij cell lysis buffer. The cell lysates were resolved onto SDS-PAGE, immunoblotted with anti-LC3 antibody (MBL, PD014), and detected by ECL.

For GFP-LC3 puncta, the cells were infected with Premo™ Autophagy Sensors (LC3B-GFP, Invitrogen) for16 hours [30]. The cells then induced autophagy as indicated, fixed with 3.7% formaldehyde solution, and were examined for GFP-LC3 immunofluorescence by confocal microscopy (FLUOVIEW FV1000-D, Olympus).

## Supporting Information

Figure S1 **A and B. FRET demonstrated that Akt-Phafin2 interaction was augmented after induction of autophagy compared to non-treated cells.** FRET (Fluorescence resonance energy transfer) assay was performed to compare the intensities of the interaction of Akt with Phafin2 before and after induction of autophagy. The results demonstrated that Akt-Phafin2 interaction was augmented after induction of autophagy by Rapamycin or HBSS treatment compared to non-treated cells (panel A. upper panel). Representative fluorescent images were shown in panels (panel A, lower panels). Please note that in the same set of experiment, combination transfection of ECFP empty vector (pCAGGS-ECFP) with Venus empty vector (pCAGGS-Venus), ECFP empty vector (pCAGGS-ECFP) with pCXN2-Venus-Phafin2, or pCXN2-ECFP-Akt2 with Venus empty vector (pCAGGS-Venus) exhibited negligible levels of FRET intensity compared to the combination transfection of pCXN2-ECFP-Akt2 and pCXN2-Venus-Phafin2. Equal levels of expression of pCXN2-ECFP-Akt2 and CXN2-Venus-Phafin2 in this experiment were confirmed by immunoblot (panel B). Method: FRET analysis was performed as essentially described elsewhere. ECFP fused Akt2 in pCXN2 (pCXN2-ECFP-Akt2) and Venus fused Phafin2 (pCXN2-Venus-Phafin2) were generated by PCR mediated subcloning. HeLa cells (ATCC) were transfected with total 3 µg of pCXN2-ECFP-Akt2 and pCXN2-Venus-Phafin2 or indicated control vectors by PEI. After 24 hrs, the cells were plated onto a 12-mm-diameter glass-base dish (Iwaki, 3911-035) in DMEM/F12 (GIBCO, 11039) supplemented with 10% FBS. 12 hours later, the cells were treated with 10 µM Rapamycin (Sigma) or washed with PBS three times and subsequently incubated in HBSS (GIBCO,14025). The cells were then imaged using an Olympus IX-71 microscope equipped with a CoolSNAP HQ cooled charge-coupled device (Photometrics, Tucson, AZ). Fluorescence intensities of FRET were measured by using the MetaMorph image processing software (Universal Imaging, Downingtown, PA). Statistical analysis was conducted using Tukey's post hoc test. The expression levels of pCXN2-ECFP-Akt2 and CXN2-Venus-Phafin2 transfected cells in this experiment were examined by immunoblot using ECL by living colors antibody (#8367-1, Clontech) or α-tubulin antibody (#T9026, Sigma).
(TIF)

Figure S2 **Positions of individual phospholipid on the PIP strip (Echlon Bioscience, P-6001) were shown.**
(TIF)

Figure S3 **PI(3)P interaction defective mutant retained the interaction with Akt in co-immunoprecipitation assays.** Method: Co-immunoprecipitation experiments were essentially performed as described previously [26], [27]. In brief, 293T cells (ATCC) were co-transfected with a total of 7.5 µg of indicated plasmids per 10 cm dish. 72 hours after transfection, cells were washed twice with ice-cold PBS and lysed with ice-cold Brij97 lysis buffer (see below) with proteinase inhibitors (Leupeptin, and AEBSF). Lysates were precleaned with protein G/protein A mixture (50% v/v, G E healthcare) for 1 hr., immunoprecipitated with anti-HA or anti-Flag antibody (or other indicated antibodies) with mouse IgG as a control, run on SDS-PAGE (8% Tris glycine gel), and immunoblotted with indicated antibodies and detected using ECL. The results were consistent at least in two independent experiments.
(TIF)

Figure S4 **A, B, and C. Phafin2-siRNA inhibited endogenous expression of Phafin2, but no effect on Akt or Phafin1 expression.** A. Phafin2-siRNA inhibited the expression of endogenous Phafin2 (top panel), but no effect on Phafin1 (second panel, negligible expression in n J774.1 murine macrophage cells) or Akt (third panel) in J774.1 murine macrophage cells. Method: J774.1 cell lines (Mouse reticulum cell sarcoma) were transfected with siRNA specific for firefly luciferase (control, Wako Nippon GENE) or Phafin2 [MSS231002 (Phafin2-1), MSS231003 (Phafin2-2): Mouse Stealth Select RNAi: Plekhf2 Stealth Select RNAi™ 3 siRNA, Invitrogen] using CUY21 Pro-vitro (NEPAGENE Co. Ltd). 72 hours later, the cells were lysed with Brij97 cell lysis buffer with proteinase inhibitors (leupeptin and AEBSF), 1 mM Na_3_VO_4_ and 10 mM NaF. 20 µg of the cell lysates were loaded onto SDS-PAGE, and immunoblotted with anti-Phafin2 (anti-rabbit polyclonal antibody), anti-Phafin1 (PAB 5534, Abnova), anti-Akt 9272, Cell Signaling), or anti-α-tubulin (T9026, DM1A, Sigma) antibodies and detected using ECL. B. Phafin2 siRNA did not affect expression levels of Akt. Method: J774.1 cell lines were transfected with siRNA specific for firefly luciferase (control, Wako Nippon GENE) or Phafin2 [MSS231002 (Phafin2-1), MSS231003 (Phafin2-2): Mouse Stealth Select RNAi: Plekhf2 Stealth Select RNAi™ 3 siRNA, Invitrogen] using CUY21 Pro-vitro (NEPAGENE Co.Ltd). 72 hours later, the cells were fixed with 3.7% formaldehyde and stained with anti-Akt antibody (2966, Cell Signaling Technology) and visualized using confocal microscopy (FLUOVIEW FV-1000-D, Olympus). White scale bar represents 10 µm. C. J774.1 murine macrophages expressed negligible levels of Phafin1 compared to the expression of Phafin2 by immunoblot. Method: 293T cells were transfected with Flag-tagged human Phafin1 or Phafin2 by calcium phosphate transfection. The cells were cultured for additional 48 hours in DMEM supplemented with 10% FBS. These transfected cells and J774.1 cells were harvested, lysed in Brij cell lysis buffer and resolved onto SDS-PAGE and immunoblotted with anti-Phafin1 antibody (PAB5534, Abnova), anti-Phafin2 antibody (polyclonal anti-Phafin2 rabbit serum), and anti-α-tubulin antibody (T9026, Sigma) and detected using ECL.
(TIF)

Figure S5 **Akt siRNA effectively inhibited its expression.** A. (Fig. 4N-Q Akt expression). J774.1 cell lines were transfected with 100 nM siRNA specific for firefly luciferase (control, Wako Nippon GENE) or Akt-siRNA (Cell Signaling, 6211) using CUY21 Pro-vitro (NEPAGENE Co. Ltd). Cells were lysed with Brij lysis buffer and resolved onto SDS gel and immunoblotted using anti Akt (upper panel) or anti α-tubulin (lower panel) antibodies and visualized by ECL. B. (Fig. 4R–U Akt expression). HeLa cells, cultured in DMEM supplemented with 10% FBS, were transfected with 100 nM Akt-siRNA (Cell Signaling, 6211S) or firefly luciferase siRNA (control, Wako Nippon GENE) as a control using CUY21 Pro-vitro (NEPAGENE Co. Ltd). Seventy two hours after transfection, cells were lysed with Brij lysis buffer and resolved onto SDS gel and immunoblotted using anti Akt (upper panel) or anti α-tubulin (lower panel) antibodies and visualized by ECL. C. (Fig. 4V Akt expression). J774.1 cell lines were transfected with 100 nM siRNA specific for firefly luciferase (control, Wako Nippon GENE) or Akt [SignalSilence Akt siRNAI (Cell Signaling, 6211)] as indicated using CUY21 Pro-vitro (NEPAGENE Co. Ltd). Seventy two hours after transfection, cells were lysed with Brij lysis buffer and resolved onto SDS gel and immunoblotted using anti Akt (upper panel) or anti α-tubulin (lower panel) antibodies and visualized by ECL.
(TIF)

Movie S1 **In BiFC, WT Phafin2 displayed granular patterns in the cytosol and perinuclear accumulation of the Akt-Phafin2 complex.** S1: After Rapamycin treatment to induce autophagy, wild type Phafin2 displayed granular pattern in the cytosol which presumably formation of the Akt-Phafin2 complex at the lysosome and perinuclear accumulation of the Akt-Phafin2 complex determined by the presence of the bright enlarged protein aggregate in BiFC analysis. Method: HeLa cells were transfected by PEI with total of 3 µg of Akt C-term Venus, and N-term Venus Phafin2 along with wild type (WT, movie S1) Phafin2 or PI(3)P interaction defective mutant Phafin2 (R53C, R171A, and R172A, movie S2) as indicated (see details in BiFC experiment of the Material and Methods in supplemental information). The cells were plated on a 12-mm-diameter glass-base dish (Iwaki, 3911-035) in DMEM/F12 (Invitrogen, 110390-021) supplemented with 10% FBS. Twenty four hours after transfection, cells were treated with 10 µM Rapamycin (Sigma) in DMEM/F12 supplemented with 10% FBS for 4 hours in a chamber box on a microscope at 37°C and analyzed using MetaMorph (Version 7.5.6, MDS Analytical Technologies).
(AVI)

Movie S2 **Mutant Phafin2 showed no granular patterns in the cytosol and perinuclear accumulation of the Akt-Phafin2 complex.** S2: By contrast, PI(3)P interacting defective mutant Phafin2 (R53C, R171A, and R172A), which did not bind to PI(3)P (Fig. 5A), displayed almost no granular patterns in the cytosol nor perinuclear accumulation of the Akt-Phafin2 complex determined by the absence of the enlarged perinuclear protein aggregates after induction of autophagy in BiFC analysis.
(AVI)
