# Supplementary figures and images for: Lysosomal Interaction of Akt with Phafin2: A Critical Step in the Induction of Autophagy
Source: PLoS One. 2014 Jan 8;9(1):e79795. doi: 10.1371/journal.pone.0079795 (PMC3885392; doi:10.1371/journal.pone.0079795)

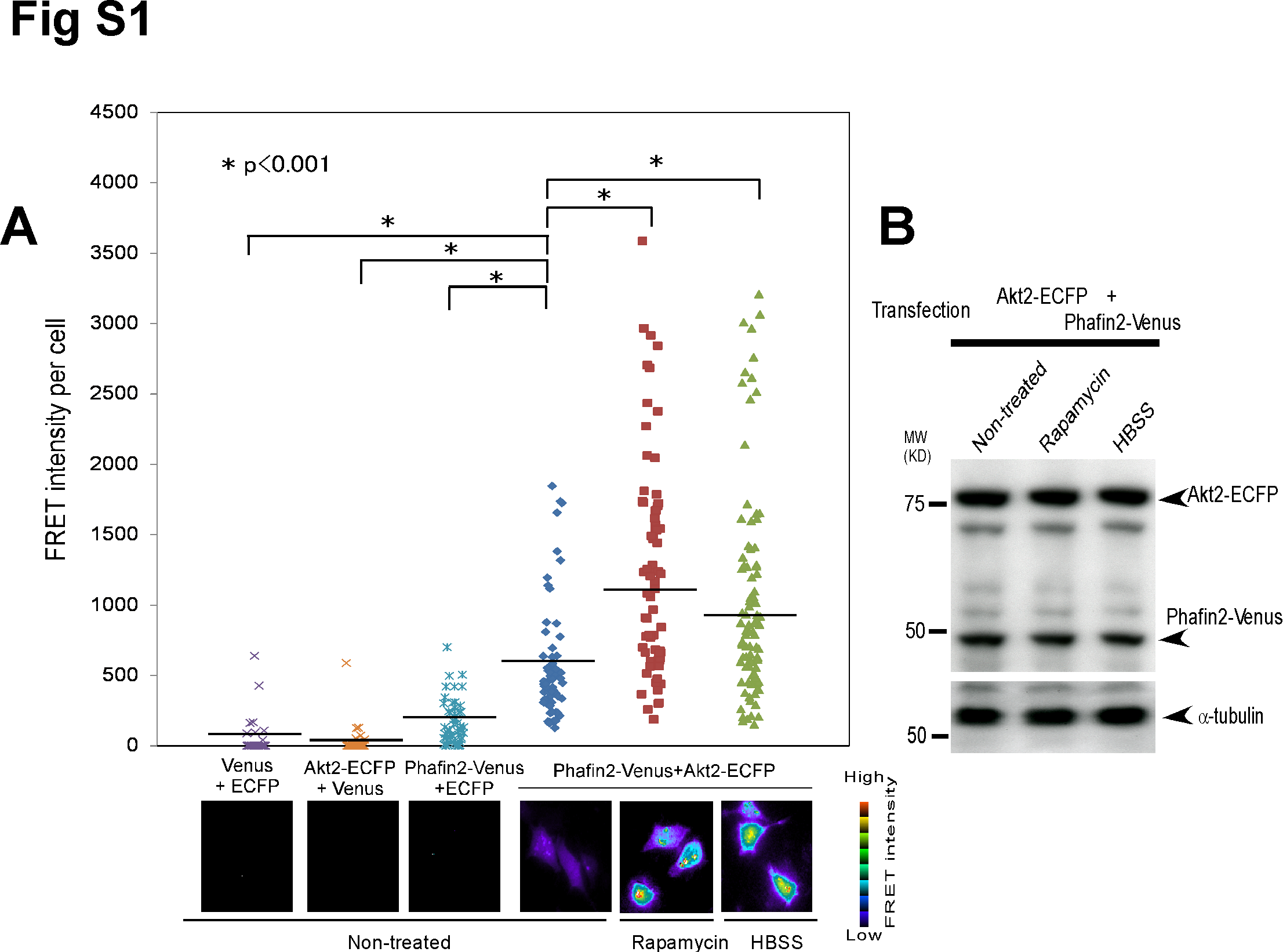

Supplement: Figure S1 — A and B. FRET demonstrated that Akt-Phafin2 interaction was augmented after induction of autophagy compared to non-treated cells. FRET (Fluorescence resonance energy transfer) assay was performed to compare the intensities of the interaction of Akt with Phafin2 before and after induction of autophagy. The results demonstrated that Akt-Phafin2 interaction was augmented after induction of autophagy by Rapamycin or HBSS treatment compared to non-treated cells (panel A. upper panel). Representative fluorescent images were shown in panels (panel A, lower panels). Please note that in the same set of experiment, combination transfection of ECFP empty vector (pCAGGS-ECFP) with Venus empty vector (pCAGGS-Venus), ECFP empty vector (pCAGGS-ECFP) with pCXN2-Venus-Phafin2, or pCXN2-ECFP-Akt2 with Venus empty vector (pCAGGS-Venus) exhibited negligible levels of FRET intensity compared to the combination transfection of pCXN2-ECFP-Akt2 and pCXN2-Venus-Phafin2. Equal levels of expression of pCXN2-ECFP-Akt2 and CXN2-Venus-Phafin2 in this experiment were confirmed by immunoblot (panel B). Method: FRET analysis was performed as essentially described elsewhere. ECFP fused Akt2 in pCXN2 (pCXN2-ECFP-Akt2) and Venus fused Phafin2 (pCXN2-Venus-Phafin2) were generated by PCR mediated subcloning. HeLa cells (ATCC) were transfected with total 3 µg of pCXN2-ECFP-Akt2 and pCXN2-Venus-Phafin2 or indicated control vectors by PEI. After 24 hrs, the cells were plated onto a 12-mm-diameter glass-base dish (Iwaki, 3911-035) in DMEM/F12 (GIBCO, 11039) supplemented with 10% FBS. 12 hours later, the cells were treated with 10 µM Rapamycin (Sigma) or washed with PBS three times and subsequently incubated in HBSS (GIBCO,14025). The cells were then imaged using an Olympus IX-71 microscope equipped with a CoolSNAP HQ cooled charge-coupled device (Photometrics, Tucson, AZ). Fluorescence intensities of FRET were measured by using the MetaMorph image processing software (Universal Imaging, Downingtown, PA). St [file pone.0079795.s001.tif]

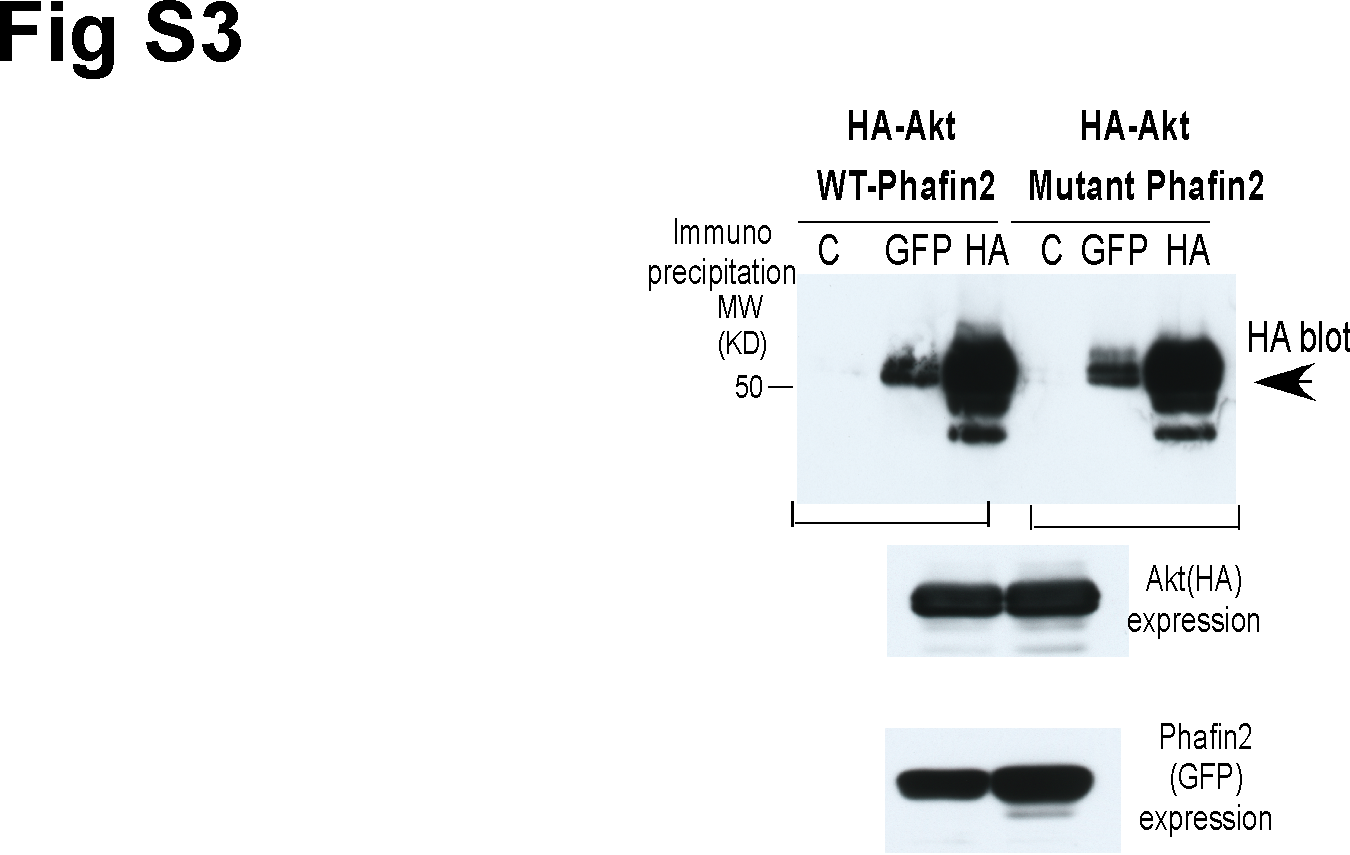

Supplement: Figure S3 — PI(3)P interaction defective mutant retained the interaction with Akt in co-immunoprecipitation assays. Method: Co-immunoprecipitation experiments were essentially performed as described previously [26], [27]. In brief, 293T cells (ATCC) were co-transfected with a total of 7.5 µg of indicated plasmids per 10 cm dish. 72 hours after transfection, cells were washed twice with ice-cold PBS and lysed with ice-cold Brij97 lysis buffer (see below) with proteinase inhibitors (Leupeptin, and AEBSF). Lysates were precleaned with protein G/protein A mixture (50% v/v, G E healthcare) for 1 hr., immunoprecipitated with anti-HA or anti-Flag antibody (or other indicated antibodies) with mouse IgG as a control, run on SDS-PAGE (8% Tris glycine gel), and immunoblotted with indicated antibodies and detected using ECL. The results were consistent at least in two independent experiments. (TIF) [file pone.0079795.s003.tif]

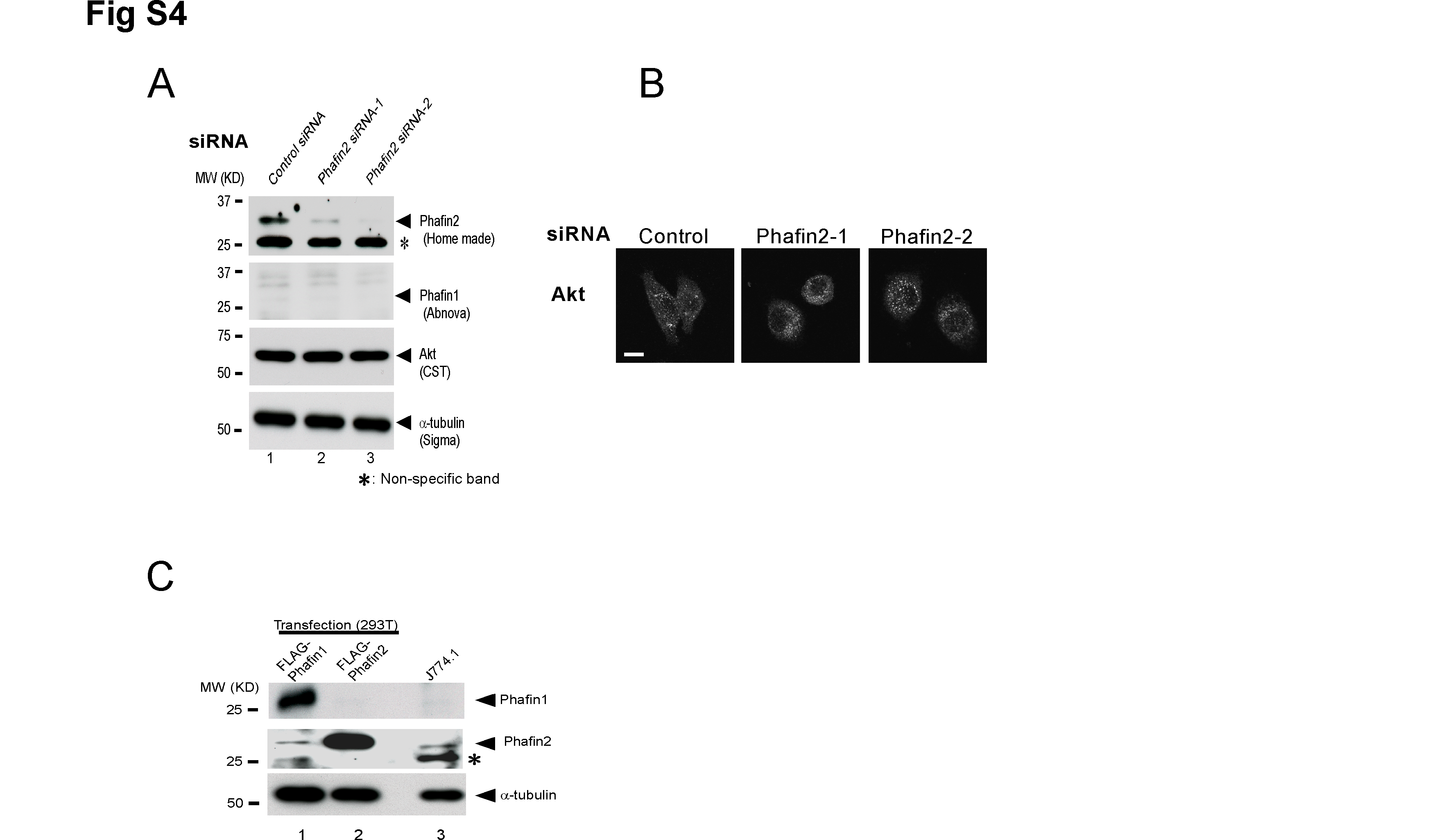

Supplement: Figure S4 — A, B, and C. Phafin2-siRNA inhibited endogenous expression of Phafin2, but no effect on Akt or Phafin1 expression. A. Phafin2-siRNA inhibited the expression of endogenous Phafin2 (top panel), but no effect on Phafin1 (second panel, negligible expression in n J774.1 murine macrophage cells) or Akt (third panel) in J774.1 murine macrophage cells. Method: J774.1 cell lines (Mouse reticulum cell sarcoma) were transfected with siRNA specific for firefly luciferase (control, Wako Nippon GENE) or Phafin2 [MSS231002 (Phafin2-1), MSS231003 (Phafin2-2): Mouse Stealth Select RNAi: Plekhf2 Stealth Select RNAi™ 3 siRNA, Invitrogen] using CUY21 Pro-vitro (NEPAGENE Co. Ltd). 72 hours later, the cells were lysed with Brij97 cell lysis buffer with proteinase inhibitors (leupeptin and AEBSF), 1 mM Na3VO4 and 10 mM NaF. 20 µg of the cell lysates were loaded onto SDS-PAGE, and immunoblotted with anti-Phafin2 (anti-rabbit polyclonal antibody), anti-Phafin1 (PAB 5534, Abnova), anti-Akt 9272, Cell Signaling), or anti-α-tubulin (T9026, DM1A, Sigma) antibodies and detected using ECL. B. Phafin2 siRNA did not affect expression levels of Akt. Method: J774.1 cell lines were transfected with siRNA specific for firefly luciferase (control, Wako Nippon GENE) or Phafin2 [MSS231002 (Phafin2-1), MSS231003 (Phafin2-2): Mouse Stealth Select RNAi: Plekhf2 Stealth Select RNAi™ 3 siRNA, Invitrogen] using CUY21 Pro-vitro (NEPAGENE Co.Ltd). 72 hours later, the cells were fixed with 3.7% formaldehyde and stained with anti-Akt antibody (2966, Cell Signaling Technology) and visualized using confocal microscopy (FLUOVIEW FV-1000-D, Olympus). White scale bar represents 10 µm. C. J774.1 murine macrophages expressed negligible levels of Phafin1 compared to the expression of Phafin2 by immunoblot. Method: 293T cells were transfected with Flag-tagged human Phafin1 or Phafin2 by calcium phosphate transfection. The cells were cultured for additional 48 hours in DMEM supplemented with 10% FBS. These transfected cells [file pone.0079795.s004.tif]

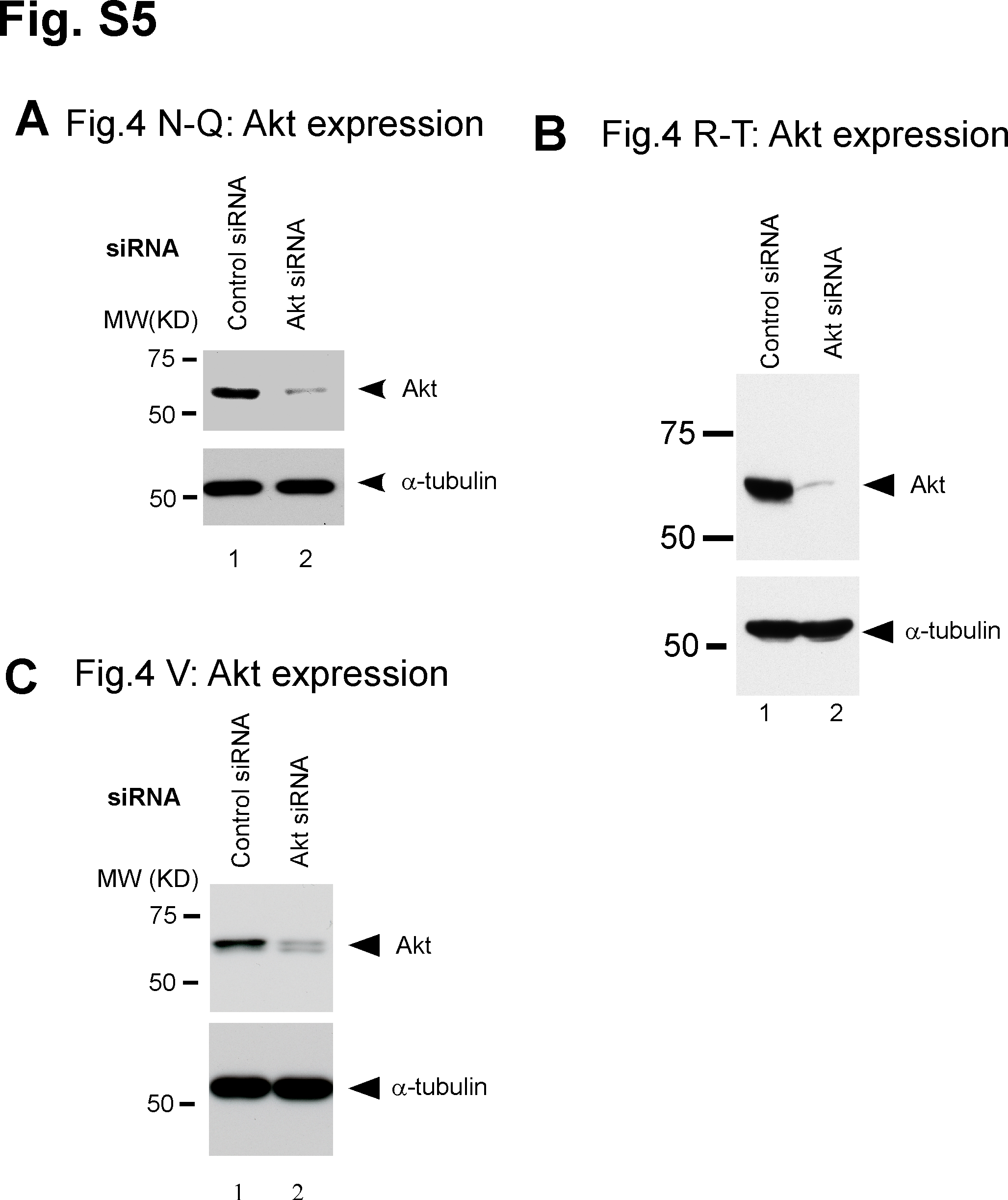

Supplement: Figure S5 — Akt siRNA effectively inhibited its expression. A. (Fig. 4N-Q Akt expression). J774.1 cell lines were transfected with 100 nM siRNA specific for firefly luciferase (control, Wako Nippon GENE) or Akt-siRNA (Cell Signaling, 6211) using CUY21 Pro-vitro (NEPAGENE Co. Ltd). Cells were lysed with Brij lysis buffer and resolved onto SDS gel and immunoblotted using anti Akt (upper panel) or anti α-tubulin (lower panel) antibodies and visualized by ECL. B. (Fig. 4R–U Akt expression). HeLa cells, cultured in DMEM supplemented with 10% FBS, were transfected with 100 nM Akt-siRNA (Cell Signaling, 6211S) or firefly luciferase siRNA (control, Wako Nippon GENE) as a control using CUY21 Pro-vitro (NEPAGENE Co. Ltd). Seventy two hours after transfection, cells were lysed with Brij lysis buffer and resolved onto SDS gel and immunoblotted using anti Akt (upper panel) or anti α-tubulin (lower panel) antibodies and visualized by ECL. C. (Fig. 4V Akt expression). J774.1 cell lines were transfected with 100 nM siRNA specific for firefly luciferase (control, Wako Nippon GENE) or Akt [SignalSilence Akt siRNAI (Cell Signaling, 6211)] as indicated using CUY21 Pro-vitro (NEPAGENE Co. Ltd). Seventy two hours after transfection, cells were lysed with Brij lysis buffer and resolved onto SDS gel and immunoblotted using anti Akt (upper panel) or anti α-tubulin (lower panel) antibodies and visualized by ECL. (TIF) [file pone.0079795.s005.tif]
